# HAX1 Augments Cell Proliferation, Migration, Adhesion, and Invasion Induced by Urokinase-Type Plasminogen Activator Receptor

**DOI:** 10.1155/2012/950749

**Published:** 2012-01-17

**Authors:** Ahmed H. Mekkawy, David L. Morris, Mohammad H. Pourgholami

**Affiliations:** Cancer Research Laboratories, Department of Surgery, St. George Hospital, University of New South Wales, Sydney, NSW 2217, Australia

## Abstract

The urokinase-type plasminogen activator receptor (uPAR) is a cell surface receptor which has a multifunctional task in the process of tumorigenesis including cell proliferation, adhesion, migration, and invasion. Many of the biological functions of uPAR necessitate interactions with other proteins. We have shown previously that uPAR interacts with HAX1 protein (HS-1-associated protein X-1). In the current study, to gain insight into the possible role of HAX1 overexpression in regulation of uPAR signal transduction pathway, several function assays were used. We found that, upon stimulation of uPAR, HAX1 colocalizes with uPAR suggesting a physiological role for HAX1 in the regulation of uPAR signal transduction. HAX1 overexpression augments cell proliferation and migration in uPAR-stimulated cells. Moreover, HAX1 over-expression augmented uPAR-induced cell adhesion to vitronectin as well as cellular invasion. Our results suggest that HAX1 over-expression may underlay a novel mechanism to regulate uPAR-induced functions in cancer cells.

## 1. Introduction

The urokinase-type plasminogen activator (uPA) receptor (uPAR) has been implicated in cell proliferation, migration, adhesion, invasion, and signal transduction apart from its role in extracellular matrix (ECM) and basement membrane proteolysis [1]. The uPAR protein consists of three domains (DI, DII, and DIII) [2]. uPAR DI is the ligand-binding site for uPA [3], whilst uPAR DII and DIII host the binding sites for other proteins such as integrins and vitronectin (Vn) [4, 5]. The active uPA consists of catalytic protease domain and uPA amino terminal fragment (uPA-ATF) [6]. uPA-ATF contains the kringle domain and the growth factor-like domain (GFD) [6]. GFD contains the binding sequence for the receptor [6].

uPA system has been shown to be involved in cell proliferation. Transfection of relatively low uPAR expressing MS-1 human pleural mesothelial cells with uPAR cDNA increased proliferation and migration *in vitro* and tumor formation *in vivo* [7]. Moreover, it has been shown that suppression of uPAR inhibits proliferation and migration of pancreatic adenocarcinoma cells via regulation of extracellular signal-regulated kinases (ERK)/p38 signaling [8]. Cells that were treated with uPA, uPA-ATF, or uPAR-devoid of domain 1 were activated, leading to their enhanced migration [9, 10]. uPA can influence cell migration by directly cleaving ECM proteins such as fibronectin [11], or by activating pro-transforming growth factor-*β* (pro-TGF-*β*) [12], and pro-hepatocyte growth factor (pro-HGF) [13]. On the contrary, the intratumoral or systemic delivery of uPA-ATF gene induced significant inhibition of angiogenesis-associated tumor growth, invasion, and metastasis of tumor cells *in vivo *[14, 15]. Furthermore, endogenous uPA-ATF expression repressed invasion and metastasis of lung cancer cells [16]. In addition, uPA-ATF has also shown to restrain the invasion of breast carcinoma [17]. Recently, soluble uPAR has been found to be involved in chondrosarcoma cell mobilization [18]. We have recently identified HAX1 (HS-1-associated protein X-1) as a novel partner of uPAR [19]. Initially, to identify a functional significance of the interaction between HAX1 and uPAR, we demonstrated the colocalization of uPAR and HAX1 in different cell lines upon stimulation of cells with different stimulants to uPAR signal transduction pathway. These stimulants included epidermal growth factor (EGF), uPA, and uPA-ATF. Subsequently, we showed for the first time that HAX1 overexpression could augment cell proliferation, migration, adhesion, and invasion induced by uPAR.

## 2. Material and Methods

### 2.1. Cell Lines, Transfection, and Reagents

The human embryonic kidney HEK293 cells stably transfected with uPAR were kindly provided by Dr. Ying Wei (University of California, San Francisco, CA, USA). The human breast cancer MDA-MB-231 and the human osteosarcoma Saos-2 cell lines were obtained from American Tissue Culture Collection (ATCC). Cells were maintained in Dulbecco's modified Eagle's medium supplemented with 10% fetal calf serum (FCS) and 1% antibiotics. The pGEM-3Zf(+)*∖*HAX1 vector was kindly provided by Dr. Maria Olsson (Göteborg University, Sahlgrenska University Hospital, Gothenburg, Sweden). Cells were transfected with recombinant or control vector in addition to GFP plasmid to control transfection efficiency. All transfections were carried out using GeneJuice (no. 70967, Novagen, Germany), according to manufacturer's instructions. Goat anti-human uPAR antibody was purchased from R&D Systems (no. AF807, USA). Rabbit anti-human HAX1 antibody (no. sc-28268) was purchased from Santa Cruz Biotechnology Inc. The recombinant analog EGF was obtained from Sigma-Aldrich (no. E-4269, USA). The recombinant human uPA was purchased from R&D systems (no. 1310-SE, USA). The recombinant human uPA-ATF was obtained from American Diagnostics (no. 146, USA).

### 2.2. Confocal Microscopy

Transfected cells with pGEM-3Zf(+)*∖*HAX1 were seeded onto sterilized glass cover slips. For the stimulation experiments, cells were serum-starved overnight, treated with 100 ng/mL EGF, uPA, or uPA-ATF for 20 min, washed with PBS, and fixed with 0.5% formaldehyde/PBS/0.1% sodium azide for 1 h. Afterwards, cells were washed and incubated in 70% ethanol for 1 h at 4°C. Fixed cells were washed, blocked with 1% BSA, and then incubated with polyclonal rabbit anti-human HAX1 and goat anti-human uPAR and primary antibodies in 1% BSA, followed by anti-rabbit FITC-conjugated and anti-goat Rhodamine phalloidin-conjugated secondary antibodies in 1% BSA. After washing, cover slips were mounted and cells analysed using confocal Olympus IX71 Laser Scanning Microscope to determine the extent of colocalization.

### 2.3. MTT Proliferation Assay

Proliferation assay was performed as previously described [20]. Briefly, 1 × 10^5^ cells/well were cultured in 96-well plates and incubated at 37°C in 5% CO_2_ incubator for 24 h. At the endpoint, cells were incubated with MTT 0.5 mg/mL for further 4 h. Resulting formazan crystals were dissolved with 100 *μ*L DMSO (dimethyl sulfoxide). Absorbance was measured at 570 nm using a 96-well plate reader.

### 2.4. Migration Assay

Effect of HAX1 on cell migration was studied using wound healing procedure as reported by Rodriguez et al. [21]. Cells were seeded into culture dishes and incubated for 24 h at 37°C. The cells were transfected with either pGEM-3Zf(+)*∖*HAX1 or pGEM-3Zf(+) empty plasmid and incubated at 37°C in 5% CO_2_ to create confluent monolayer. A scratch was created manually by scrapping the cell monolayer with yellow pipette tip. After washing, 5 mL of culture media with 2% FCS was added to culture media. The first image was taken by using marks on the culture dish as a reference point. Cells were stimulated with EGF, uPA, or uPA-ATF 100 ng/mL media or left without stimulation as a control and incubated in the CO_2_ incubator for 18 h at 37°C, and then the second image was acquired. These images were analysed quantitatively by measuring the distance of the wounded region migrated by cells in pixels.

### 2.5. Cell Adhesion Assay

The assay was performed as previously described with minor modifications [22]. Briefly, 96-well dishes were precoated with 2 *μ*g/mL Vn or heat-denatured BSA (Sigma-Aldrich) overnight at 4°C. Wells were rinsed with PBS and incubated with 2% heat-denatured BSA to block any uncoated areas. Cells (1.5 × 10^5^/well) were seeded in the coated wells and incubated for 2 h at 37°C. After washing the attached cells were fixed with methanol/acetone and stained with 0.1% crystal violet. The stain was eluted using acetic acid/methanol/water and absorbance measured at 595 nm with a 96-well plate reader.

### 2.6. Cell Invasion Assay

Cell invasion was measured using 24-well Transwell system with polycarbonate membranes of 8 mm pore size. The membranes were coated with 20 *μ*g/mL collagen IV at 4°C overnight. Transfected cells (2 × 10^5^/well) were seeded onto the upper-side chambers in 0.2 mL of serum-free DMEM medium and 0.6 mL of the same medium containing 1% FCS was added to the lower chamber. The cells were allowed to adhere for 1 h. Then chemotaxis was induced by the addition of 100 ng/mL EGF, uPA, or uPA-ATF to the lower chamber. Media containing 10% FCS or 1% FCS were used as positive and negative controls, respectively. After 18 h, cells that invaded through the membrane to the lower surface were Giemsa stained and counted in five different fields under the light microscope.

### 2.7. Statistical Analysis

Data is expressed as the mean ± S.D. and where appropriate, the Student's *t-*test. Results were considered statistically significant when *P* < 0.05.

## 3. Results

### 3.1. HAX1 Colocalized with uPAR upon Stimulation of Cells with EGF, uPA, and uPA-ATF

**Figure 1:**
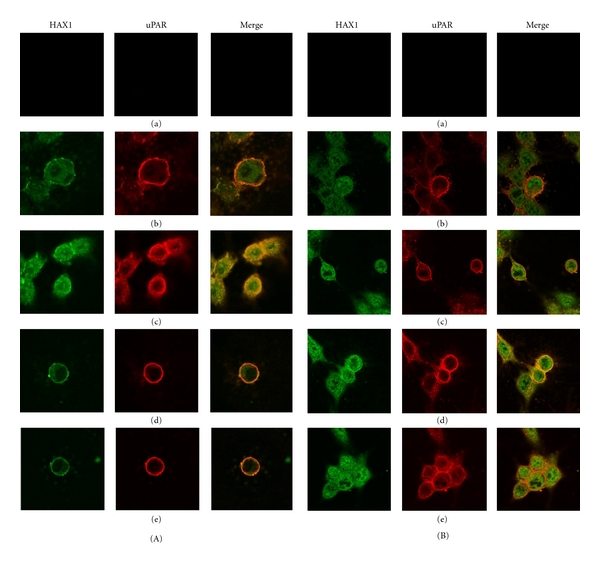
HAX1 colocalizes with uPAR upon stimulation of cells with uPA and uPA-ATF. (A) HEK293/uPAR and (B) MDA-MB-231 cells were transfected with pGEM-3Zf(+)*∖*HAX1, kept as negative control (a) or serum-starved overnight (b) and then treated with 100 ng/mL of either EGF (c), uPA, (d) or uPA-ATF (e) for 20 min. Cells were fixed and then immunostained with antibodies against uPAR (red) and HAX1 (green), and colocalization appeared as yellow colour. These cells were analysed using confocal laser scanning microscope and 60x oil immersion lens (final magnification 600x).

uPA binding to uPAR triggers both proteolysis of ECM and signal transduction. Immunofluorescence studies were performed to investigate the cellular distribution of HAX1 and its localization with uPAR following stimulation of cells with EGF, uPA, or uPA-ATF. HEK293/uPAR and MDA-MB-231 cells transfected with HAX1 were employed for this experiment (Figure 1). In actively proliferating cells cultured in growth media, HAX1 was located in the cytoplasm. However, uPAR was primarily localized on the cell membrane and in the cytoplasm. In cells cultured in serum-starved media, HAX1 colocalization with uPAR was diminished (Figure 1). A subset of HAX1 was found to colocalize with uPAR upon stimulation of cells with EGF, uPA, or uPA-ATF (Figure 1), suggesting a physiological role for HAX1 in the regulation of uPAR signal transduction. Based on this observation along with our finding that uPAR interacts with HAX1, we decided to investigate the role of HAX1 as regulator of uPAR signal transduction pathway in cells stimulated with EGF, uPA, and uPA-ATF using different function assays.

### 3.2. HAX1 Is Involved in uPAR-Induced Cell Proliferation

**Figure 2:**
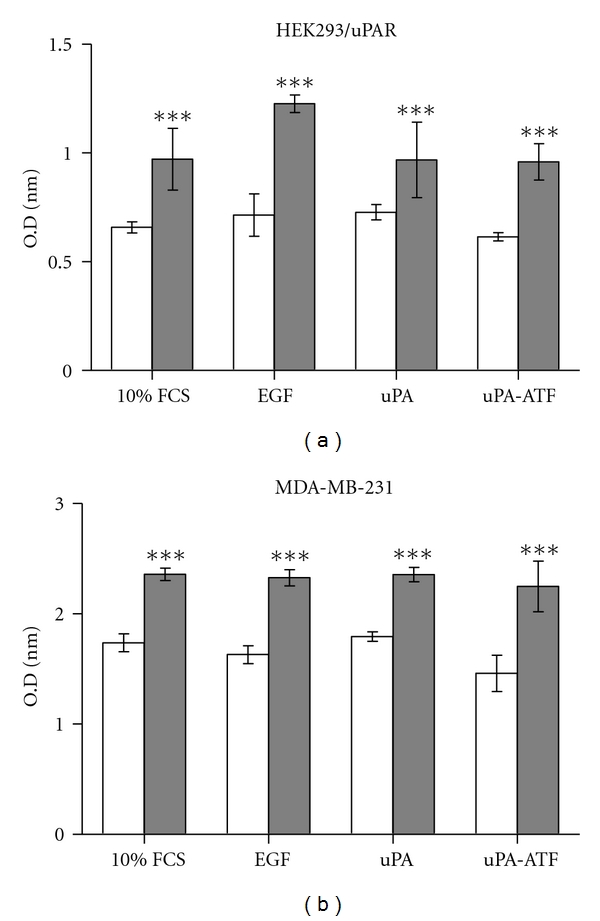
HAX1 overexpression augments HEK293/uPAR and MDA-MB-231 cell proliferation in uPAR-stimulated cells. (a) HEK293/uPAR. (b) MDA-MB-231 cells were transfected with pGEM-3Zf(+) (light bars) or pGEM-3Zf(+)*∖*HAX1 (dark bars). Cells were exposed to 10% FCS alone or with 100 ng/mL of EGF, uPA, or uPA-ATF. Cell proliferation was measured using MTT assay. Data is expressed as mean ± S.E of optic density (OD) readings; *** represents *P* < 0.001 using unpaired Student's *t*-test.

To demonstrate the physiological impact of HAX1 overexpression on uPAR-induced cell proliferation, MTT proliferation assay was performed using HEK293/uPAR and MDA-MB-231 cells transfected with pGEM-3Zf(+)*∖*HAX1 or pGEM-3Zf(+) empty plasmid. Cells were stimulated with 100 ng/mL of EGF, uPA, or uPA-ATF, while other cells were left without stimulation as a control group. Proliferation of HEK293/uPAR cells transfected with pGEM-3Zf(+)*∖*HAX1 compared to cells transfected with vector plasmid was significantly increased (*P* < 0.001) in control group (10% FCS-treated cells) (Figure 2). Proliferation of cells transfected with pGEM-3Zf(+)*∖*HAX1 and stimulated with EGF, uPA, or uPA-ATF led to profound increase in cell proliferation (*P* < 0.001) when compared to pGEM-3Zf(+)-transfected cells.

### 3.3. Overexpression of HAX1 Increases uPAR-Induced Cell Migration

**Figure 3:**
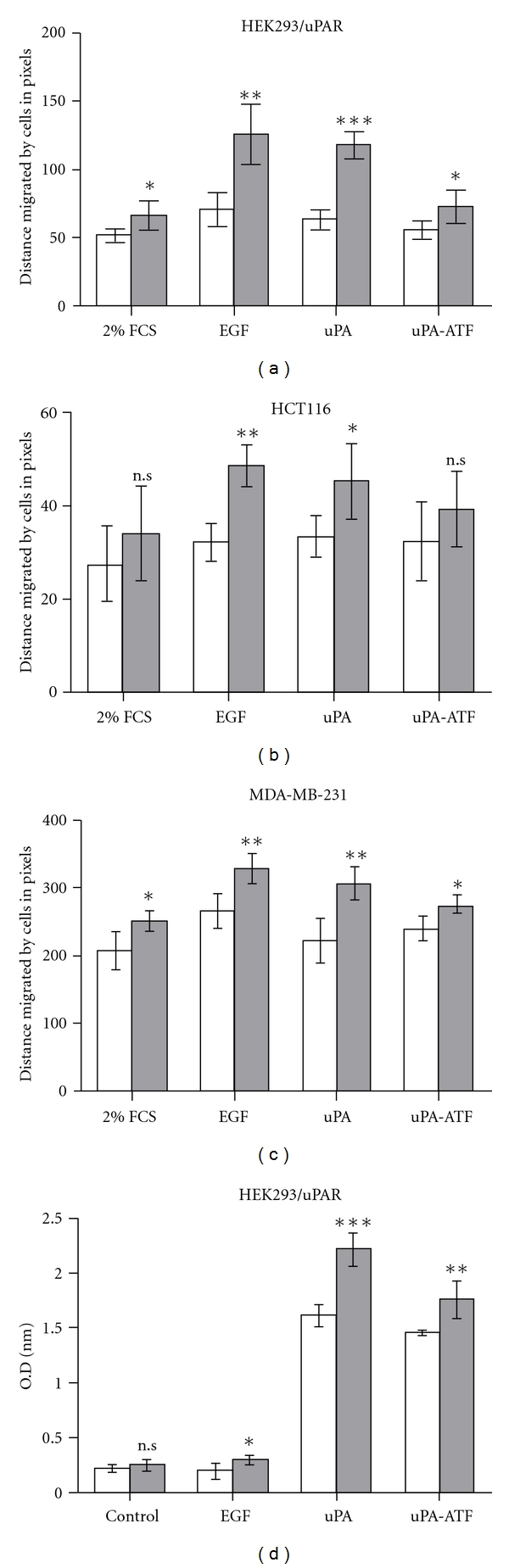
HAX1 overexpression increases cell migration and adhesion in uPAR-stimulated cells. (a) HEK293/uPAR, (b) HCT116, and (c) MDA-MB-231 cells were transfected with pGEM-3Zf(+) (light bars) or pGEM-3Zf(+)*∖*HAX1 (dark bars) and examined using wound healing assay. A monolayer of confluent cells was scratched with a 200 *μ*L pipette tip and the closure of the scratch was observed in the presence of either 10% FCS, 1% FCS, or 100 ng/mL of EGF, uPA, or ATF. Photographs were taken immediately and after 18 h of creating the scratch. Cell migration was assessed by measuring the distance migrated by cells in pixels in these 18 h. (d) HAX1 augments HEK293/uPAR cell adhesion. 1.5 × 10^5^ cells/well transfected with pGEM-3Zf(+) (light bars) or pGEM-3Zf(+)*∖*HAX1 (dark bars) were seeded in precoated 96-well plate with 2 *μ*g/mL Vn or heat-denatured BSA, treated with 100 ng/mL of EGF, uPA, or uPA-ATF, and incubated for 2 h at 37°C. After washing, the attached cells were fixed with methanol/acetone and stained with 0.1% crystal violet. The stain was eluted using acetic acid/methanol/water and absorbance measured at O.D_595_. Bars are mean ± S.E using unpaired Student's *t-*test (^n.s^ *P* > 0.05; *P < 0.05; **P < 0.01; ***P < 0.001).

Previous reports showed that uPAR promotes cell migration [23, 24]. To demonstrate the physiological impact of HAX1 overexpression on uPAR-induced cell migration, wound healing assays were performed using HEK293/uPAR, HCT116, and MDA-MB-231 cells transfected with HAX1. As shown in Figures 3(a)–3(c), migration of transfected HEK293/uPAR cells with pGEM-3Zf(+)*∖*HAX1 was on the whole greater than cells transfected with pGEM-3Zf(+) empty plasmid. In unstimulated cells, the transfection and overexpression of HAX1 caused significant (*P* < 0.05) increase of cell migration. After stimulation with EGF and uPA, the increase of cell migration was significant (*P* < 0.01) in cells transfected with HAX1 compared to cells transfected with empty plasmid. Similarly, stimulation of HAX1-transfected cells with uPA-ATF caused significant increase (*P* < 0.05) in cell migration. The results obtained from MDA-MB-231 were almost identical to those of HEK293/uPAR cell line. Stimulation of MDA-MB-231 cells with uPA caused significant increase (*P* < 0.01) of cell migration in cells transfected with HAX1 compared to cells transfected with empty plasmid.

### 3.4. HAX1 Augments uPAR-Induced Cell Adhesion

Adhesion of cells to extracellular matrix protein Vn is an important event in cancer progression and metastasis. Using cell adhesion assay, we investigated the possibility that cell adhesion to Vn may be enhanced in response to HAX1 overexpression upon stimulation with EGF, uPA, and uPA-ATF. As presented in Figure 3(d), adhesion to Vn of HEK293/uPAR cells transfected with pGEM-3Zf(+)*∖*HAX1 compared to cells transfected with vector plasmid was nonsignificant (*P* > 0.05) in control group. However, upon treatment with EGF, the adhesion of cells transfected with pGEM-3Zf(+)*∖*HAX1 was increased (*P* < 0.05) in comparison to those transfected with pGEM-3Zf(+) empty plasmids. Adhesion of cells transfected with pGEM-3Zf(+)*∖*HAX1 and treated with uPA was significantly increased (*P* < 0.001) when compared to pGEM-3Zf(+)-transfected cells. Cells treated with uPA-ATF significantly increased (*P* < 0.01) adhesion of cells transfected with HAX1. These results reveal that HAX1 overexpression augments uPAR-induced cell adhesion to extracellular matrix Vn.

### 3.5. Overexpression of HAX1 Induces uPAR-Dependant Cell Invasion

**Figure 4:**
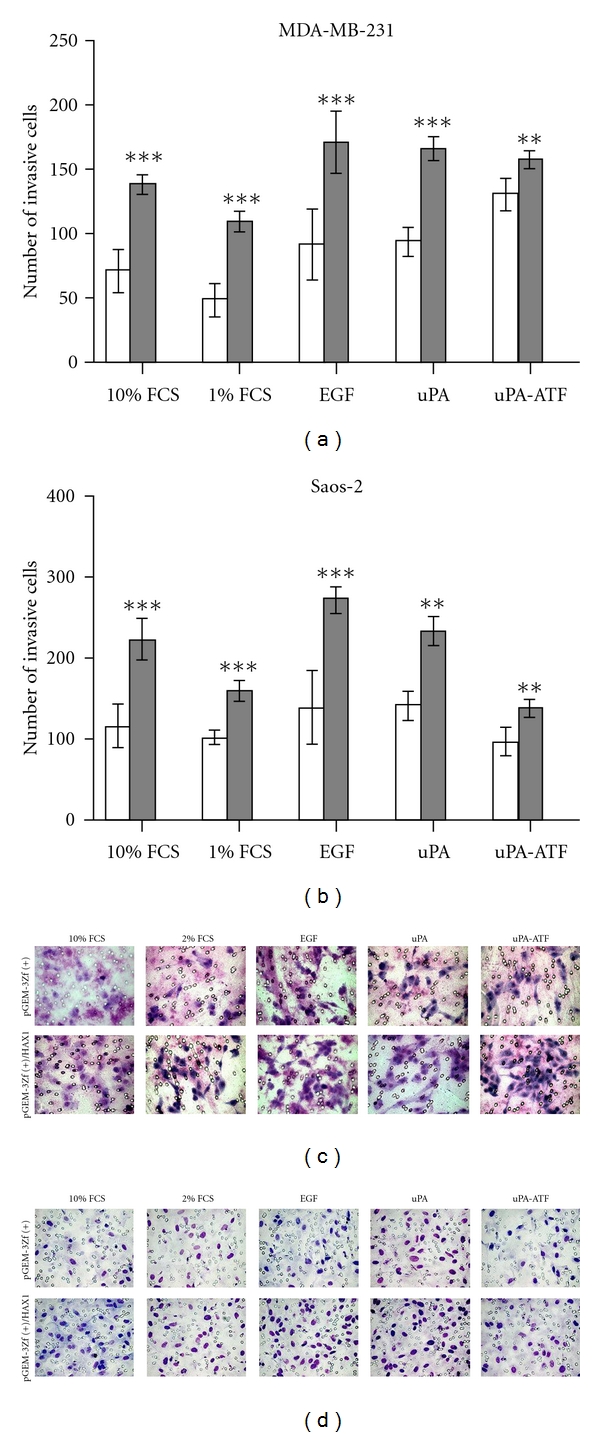
HAX1 induces cell invasion in uPAR-stimulated tumor cells. (a) MDA-MB-231 and (b) Saos-2 cells were transfected with pGEM-3Zf(+) (light bars) or pGEM-3Zf(+)*∖*HAX1 (dark bars) and placed in Transwell inserts and were exposed to either 10% FCS, 1% FCS, or 100 ng/mL of EGF, uPA, or ATF. Cell migration was assessed by counting stained (c) MDA-MB-231 and (d) Saos-2 cells on the membrane after 18 h. Data is expressed as mean ± S.E. of O.D readings (*n* = 5) using unpaired Student's *t-*test (^n.s^ *P* > 0.05; *P < 0.05; **P < 0.01; ***P < 0.001).

To determine whether HAX1 overexpression induces uPAR-induced invasion in MDA-MB-231 and Saos-2 cells, we carried out *in vitro* cell invasion assays. EGF and uPA increased cellular invasiveness of MDA-MB-231 and Saos-2 cells transfected with pGEM-3Zf(+) (Figure 4). However, the catalytically inactive uPA-ATF caused suppression of cell invasiveness (Figure 4). Compared to control pGEM-3Zf(+)-transfected cells, these cells transfected with pGEM-3Zf(+)*∖*HAX1 had significantly increased their invasive capacity (Figure 4). This was reflected in the significant increase (*P* < 0.001) in the number of invasive cells expressing HAX1 upon stimulation with EGF. However, stimulation with uPA and its catalytically inactive uPA-ATF caused a significant increase (*P* < 0.01) of cells invasiveness.

## 4. Discussion

Expression of uPAR has been shown to affect several cellular processes including proliferation, migration, adhesion, as well as invasion, and its expression has been associated with malignancy of cancers [25]. Since uPAR lacks a transmembrane domain and only attached with membrane by an extracellular glycosyl phosphatidylinositol (GPI) anchor, it uses the interaction with other partner proteins to activate signal transduction [26]. Several studies have shown that transmembrane receptors and cytoplasmic signaling proteins form a complex with or contribute in uPAR-induced signalling [25]. The identification of new interacting proteins which may bind to uPAR will allow us to better understand its complex and important role in cancer progression. Recently, we identified HAX1 as a partner protein of uPAR using yeast two-hybrid, GST-pull down, coimmunoprecipitation assays and confocal microscopy [19]. HAX1 has been found to be overexpressed in breast cancer, lung cancer, and melanoma [27, 28], although the exact molecular mechanism by which overexpression of HAX1 may provide an oncogenic role needs to be evaluated. Our preliminary data indicates that both HAX1 and uPAR are simultaneously increased in aggressive (e.g., MDA-MB-231 and PC3) but not in nonaggressive (e.g., MCF-7) cancer cells. We proposed that one of these may indeed be via its amplification of uPAR signal transduction. To gain insight into the uPAR-induced functions regulated by HAX1, we stimulated uPAR with uPA and uPA-ATF as well as EGF. The EGF receptor (EGFR) selectively has been found to cooperate with uPAR to mediate mitogenesis [29]. Our results showed profound colocalization between uPAR and HAX1 in cytoplasm of both serum-starved and stimulated cells. This was interesting as HAX1 appears to be predominantly localized to cytoplasm compartments [30], while uPAR is located in the cell membrane and cytoplasm in normal and neoplastic tissues [31, 32].

HAX1 has been also found to interact with several proteins implicated in the regulation of cell migration such as cortactin (an F-actin-associated protein) [33], the hairpin element present in the 3′UTR of the transcript of vimentin (a cytoskeletal protein) [34]. The presence of a quaternary complex consisting of HAX1, G*α*13, Rac, and cortactin indicates the role of HAX1 in the regulation of cytoskeletal components involved in cell movement. Since the expression of HAX1 potentiates G*α*13-mediated cell movement, silencing of endogenous HAX1 with HAX1-specific siRNAs significantly reduces G*α*13-mediated cell migration. These findings, together with the observation that HAX1 is overexpressed in metastatic tumors and tumor cell lines, suggest a role for HAX1/G*α*13 association in tumor metastasis [35]. In another study, HAX1 has been shown to bind to the integrin *α*v*β*6, an integrin linked to the aggressive invasive behavior of carcinoma cell and poor clinical prognosis in cancer patients [36]. Reduction of HAX1 levels by siRNA suppressed *α*v*β*6-dependent migration and invasion through clathrin-mediated endocytosis of *α*v*β*6. Following the silencing of HAX1 expression, the impaired migration was independent of apoptotic events but operated through *α*v*β*6 endocytosis [36]. In a recent study, HAX1 has been found to interact with several other proteins as a novel integrin-linked kinase (ILK) confirming the contribution of HAX1 in the integrin signaling pathway [37]. The role of HAX1 in inhibition of apoptosis and regulation of cell migration, the two critical processes in carcinogenesis and tumor metastasis, was supported by the fact that HAX1 is highly expressed in different types of human cancers [27, 36]. In the current study, we showed that HAX1 overexpression augments uPAR-induced cell proliferation, migration, adhesion, and invasion. These processes are crucial in tumor progression and metastasis. uPA binding to uPAR focuses plasmin-mediated ECM degradation to the leading edge of migrating cells and thereby facilitates cellular penetration of tissue boundaries [38]. uPAR interactions with Vn and integrins modify the strength of cellular adhesion [39, 40]. Laminin-5, a marker of invading cancer cells in some human carcinomas, is coexpressed with uPAR in budding cancer cells in colon adenocarcinomas [41]. In addition, there have been shown integrin-mediated cell adhesion to laminin-5 potentiates cell invasion and matrix metalloproteinase (MMP) production gastric carcinoma [42]. So, it is likely that activation of uPAR by HAX1 coordinates with integrins to promote cell adhesion to extracellular matrix proteins including vitronectin and laminin-5. Moreover, uPA has been found to initiate ECM proteolysis which is involved in many processes in which cell migration occurs, including tumor cell invasion [43] and monocyte infiltration [38]. Furthermore, uPA has been shown to stimulate adhesion and chemotactic movement of myeloid cells [44], to induce cell migration in human ECs [45], independently of its proteolytic activity.

In conclusion, our results showed that HAX1 overexpression augments cell proliferation and migration in uPAR-stimulated cells. Moreover, HAX1 overexpression augmented uPAR-induced cell adhesion to vitronectin as well as cellular invasion. These results suggest a novel mechanism for regulation of uPAR-induced cellular functions which may extend our understanding of the precise role of uPAR in cancer molecular biology. Further work to identify the exact downstream signal transduction pathway by which HAX1 modulates these functions is currently under investigation in our laboratories.

## Abbreviations

ATCC: American tissue culture collection
DMSO: Dimethyl sulfoxide
ECM: Extracellular matrix
EGF: Epidermal growth factor
EGFR: EGF receptor
ERK: Extracellular signal-regulated kinases
FCS: Fetal calf serum
GFD: Growth factor-like domain
GPI: Glycosyl phosphatidylinositol
HAX1: HS-1 associated protein X-1
ILK: Integrin-linked kinase
MMP: Matrix metalloproteinase
pro-HGF: Prohepatocyte growth factor
pro-TGF-*β*: Protransforming growth factor-*β*
uPA: Urokinase-type plasminogen activator
uPA-ATF: uPA amino terminal fragment
uPAR: uPA receptor
Vn: Vitronectin.
